# Risk prediction for estrogen receptor-specific breast cancers in two large prospective cohorts

**DOI:** 10.1186/s13058-018-1073-0

**Published:** 2018-12-03

**Authors:** Kuanrong Li, Garnet Anderson, Vivian Viallon, Patrick Arveux, Marina Kvaskoff, Agnès Fournier, Vittorio Krogh, Rosario Tumino, Maria-Jose Sánchez, Eva Ardanaz, María-Dolores Chirlaque, Antonio Agudo, David C. Muller, Todd Smith, Ioanna Tzoulaki, Timothy J. Key, Bas Bueno-de-Mesquita, Antonia Trichopoulou, Christina Bamia, Philippos Orfanos, Rudolf Kaaks, Anika Hüsing, Renée T. Fortner, Anne Zeleniuch-Jacquotte, Malin Sund, Christina C. Dahm, Kim Overvad, Dagfinn Aune, Elisabete Weiderpass, Isabelle Romieu, Elio Riboli, Marc J. Gunter, Laure Dossus, Ross Prentice, Pietro Ferrari

**Affiliations:** 1 Nutritional Methodology and Biostatistics Group, Nutrition and Metabolism Section, International Agency for Research on Cancer, 150 cours Albert Thomas, 69372 Lyon Cedex 08, France; 2 Public Health Sciences Division, Fred Hutchinson Cancer Research Center, Seattle, USA; 3 Breast and Gynaecologic Cancer Registry of Côte d’Or, Georges-François Leclerc Comprehensive Cancer Care Centre, Dijon, France; 4 EA 4184, Medical School, University of Burgundy, Dijon, France; 5 CESP, INSERM U1018, Univ. Paris-Sud, UVSQ, Université Paris-Saclay, Villejuif, France; 6 Gustave Roussy, Villejuif, France; 7 Epidemiology and Prevention Unit, Fondazione IRCCS Istituto Nazionale dei Tumori, Milan, Italy; 8 Cancer Registry and Histopathology Department, “Civic-M. P.Arezzo” Hospital, ASP, Ragusa, Italy; 9 Escuela Andaluza de Salud Pública, Instituto de Investigación Biosanitaria ibs. GRANADA, Hospitales Universitarios de Granada/ Universidad de Granada, Granada, Spain; 10 CIBER de Epidemiología y Salud Pública (CIBERESP), Madrid, Spain; 11 Navarra Public Health Institute, Pamplona, Spain; 12 IdiSNA, Navarra Institute for Health Research, Pamplona, Spain; 13 CIBER de Epidemiología y Salud Pública (CIBERESP), Madrid, Spain; 14 Department of Epidemiology, Regional Health Council, IMIB-Arrixaca, Murcia, Spain; 15 Department of Health and Social Sciences, Universidad de Murcia, Murcia, Spain; 16 Unit of Nutrition and Cancer. Cancer Epidemiology Research Program, Catalan Institute of Oncology-IDIBELL. L’Hospitalet de Llobregat, Barcelona, Spain; 17 Department of Epidemiology & Biostatistics, School of Public Health, Imperial College London, London, UK; 18 Cancer Epidemiology Unit, Nuffield Department of Population Health, University of Oxford, Oxford, UK; 19 Department for Determinants of Chronic Diseases, National Institute for Public Health and the Environment, Bilthoven, The Netherlands; 20 Department of Gastroenterology and Hepatology, University Medical Centre, Utrecht, The Netherlands; 21 Department of Social & Preventive Medicine, Faculty of Medicine, University of Malaya, Kuala Lumpur, Malaysia; 22 Hellenic Health Foundation, Athens, Greece; 23 WHO Collaborating Center for Nutrition and Health, Unit of Nutritional Epidemiology and Nutrition in Public Health, Department of Hygiene, Epidemiology and Medical Statistics, School of Medicine, National and Kapodistrian University of Athens, Athens, Greece; 24 Division of Cancer Epidemiology, German Cancer Research Center, Heidelberg, Germany; 25 Department of Population Health, New York University School of Medicine, New York, USA; 26 Department of Environmental Medicine, New York University School of Medicine, New York, USA; 27 Perlmutter Cancer Center, New York University School of Medicine, New York, USA; 28 Department of Public Health and Clinical Medicine, Nutritional Research, Umeå University, Umeå, Sweden; 29 Department of Surgical and Perioperative Sciences, Umeå University, Umeå, Sweden; 30 Section for Epidemiology, Department of Public Health, Aarhus University, Aarhus, Denmark; 31 Department of Cardiology, Aalborg University Hospital, Aalborg, Denmark; 32 Department of Nutrition, Bjørknes University College, Oslo, Norway; 33 Department of Research, Cancer Registry of Norway, Institute of Population-Based Cancer Research, Oslo, Norway; 34 Department of Medical Epidemiology and Biostatistics, Karolinska Institutet, Stockholm, Sweden; 35 Genetic Epidemiology Group, Folkhälsan Research Center, Helsinki, Finland; 36 Department of Community Medicine, University of Tromsø, The Arctic University of Norway, Tromsø, Norway; 37 Nutritional Epidemiology Group, Nutrition and Metabolism Section, International Agency for Research on Cancer, Lyon, France; 38 Biomarkers Group, Nutrition and Metabolism Section, International Agency for Research on Cancer, Lyon, France

**Keywords:** Breast cancer, Risk prediction, Estrogen receptor, Prospective cohort, EPIC, WHI

## Abstract

**Background:**

Few published breast cancer (BC) risk prediction models consider the heterogeneity of predictor variables between estrogen-receptor positive (ER+) and negative (ER-) tumors. Using data from two large cohorts, we examined whether modeling this heterogeneity could improve prediction.

**Methods:**

We built two models, for ER+ (Model_ER+_) and ER- tumors (Model_ER-_), respectively, in 281,330 women (51% postmenopausal at recruitment) from the European Prospective Investigation into Cancer and Nutrition cohort. Discrimination (*C-*statistic) and calibration (the agreement between predicted and observed tumor risks) were assessed both internally and externally in 82,319 postmenopausal women from the Women’s Health Initiative study. We performed decision curve analysis to compare Model_ER+_ and the Gail model (Model_Gail_) regarding their applicability in risk assessment for chemoprevention.

**Results:**

Parity, number of full-term pregnancies, age at first full-term pregnancy and body height were only associated with ER+ tumors. Menopausal status, age at menarche and at menopause, hormone replacement therapy, postmenopausal body mass index, and alcohol intake were homogeneously associated with ER+ and ER- tumors. Internal validation yielded a *C*-statistic of 0.64 for Model_ER+_ and 0.59 for Model_ER-_. External validation reduced the *C*-statistic of Model_ER+_ (0.59) and Model_Gail_ (0.57). In external evaluation of calibration, Model_ER+_ outperformed the Model_Gail_: the former led to a 9% overestimation of the risk of ER+ tumors, while the latter yielded a 22% underestimation of the overall BC risk. Compared with the treat-all strategy, Model_ER+_ produced equal or higher net benefits irrespective of the benefit-to-harm ratio of chemoprevention, while Model_Gail_ did not produce higher net benefits unless the benefit-to-harm ratio was below 50. The clinical applicability, i.e. the area defined by the net benefit curve and the treat-all and treat-none strategies, was 12.7 × 10^− 6^ for Model_ER+_ and 3.0 × 10^− 6^ for Model_Gail_.

**Conclusions:**

Modeling heterogeneous epidemiological risk factors might yield little improvement in BC risk prediction. Nevertheless, a model specifically predictive of ER+ tumor risk could be more applicable than an omnibus model in risk assessment for chemoprevention.

## Background

Breast cancer (BC) screening and chemoprevention strategies should prioritize women who are expected to benefit from the interventions. Risk prediction models could be useful assessment tools to facilitate this strategy, as long as the models themselves possess sufficient predictive power. So far, more than 20 risk prediction models have been developed for BC since the first model developed by Gail in 1989 [1, 2]. Initially, the Gail model (hereinafter referred to as Model_Gail_) was based on age, age at menarche and at first live birth, previous breast biopsy, and family history of BC, yielding moderate discriminatory power (*C*-statistic) of 0.58 in external validations [3, 4]. New predictors, such as breast density, hormone replacement therapy (HRT), anthropometric measures, and lifestyle factors (e.g. alcohol intake), were continuously introduced into the succeeding models, resulting in marginal improvements in prediction [5].

BC comprises etiologically distinct subtypes defined by molecular factors. Hormonal and reproductive factors, such as elevated circulating sex hormone levels, early menarche, delayed childbirth, and nulliparity, are only or are more strongly related to increased risks of subtypes expressing estrogen receptor (ER+) and progesterone receptor (PR+) [6]. Further, ER+ breast tumors respond more favorably to hormone therapy than ER-/PR- tumors [6–8]. It has been hypothesized that combining etiologically distinct subtypes as one single outcome undermines BC prediction [9]. However, most of the published BC risk prediction models are omnibus models and only one model differentiates risk associations by hormone receptor status [10].

In the current analysis, using data from the European Prospective Investigation into Cancer and Nutrition (EPIC) and the Women’s Health Initiative (WHI) study in the USA, we examined whether modeling heterogeneous risk associations by ER status, which entails building ER-specific risk prediction models, could yield better prediction of BC risk.

## Methods

### Study population for model derivation and internal validation

The study population for model derivation consisted of women recruited into the EPIC cohort from 1992 to 2000 in 10 European countries (Norway, Sweden, Denmark, the UK, the Netherlands, Germany, France, Spain, Italy, and Greece) [11, 12]. Women with one or more of the following characteristics were excluded: (1) < 40 or > 70 years of age at recruitment (*n* = 49,410); (2) diagnosed with cancer before recruitment (*n* = 39,760); and (3) no information on censoring date and/or disease status (*n* = 142). All women recruited in the study center of Malmö, Sweden were also excluded due to lack of information on ER status for all BC diagnoses (*n* = 14,396). After these exclusions, 281,330 women (51% postmenopausal at recruitment) were included in the analysis.

### Study population for external validation

The WHI study was launched in 1993 and recruited 161,808 postmenopausal women aged 50–79 years into either an observational study or one of the three clinical trials that tested the health effects of HRT, a low-fat diet, and calcium-vitamin D supplementation, respectively [13]. For the purpose of the present study, we excluded non-Caucasian women (*n* = 28,267), women in the HRT trial (*n* = 27,347), women who had mastectomy or a history of cancer at recruitment (*n* = 16,501), and women with incomplete information on the risk factors considered in our models (*n* = 29,431), resulting in a validation population of 82,319 women.

All women in the EPIC and WHI studies provided written informed consent. In the WHI study, Human Subjects Committee approval at each participating institution was provided. The present study was approved by the Ethical Review Board of the International Agency for Research on Cancer (Lyon, France).

### Risk factors and disease outcomes

Among the most frequently included predictors in current BC risk prediction models [5], the following variables were available in EPIC and WHI, and were therefore included in this study: menopausal status, age at menopause, age at menarche, duration of HRT, duration of breastfeeding, full-term pregnancy (FTP), number of FTPs, age at first FTP, body height, body mass index (BMI), interaction between BMI and menopausal status, alcohol intake, and country. Table 5 in Appendix provides the coding of these predictor variables. We retained all the women for analysis and handled the missing values by five-time multiple imputations with chained equations [14]. Three predictor variablesin the Gail model were not included in our models, i.e. family history of BC in first-degree relatives, previous breast biopsy, and history of atypical hyperplasia. In the EPIC study, family history of BC was only available for 49% of women, while information on previous breast biopsy and history of atypical hyperplasia were not collected.

Sensitivity analyses that included effect modification of parity by menopausal status in the EPIC study showed no evidence of statistically significant interactions. Similarly, no effect modifications were observed for HRT by BMI and breastfeeding by parity. These interactions were hence not retained further.

Incident BC diagnoses among the EPIC women were ascertained through national cancer registries or a combination of health insurance records, pathology registries, and regular questionnaire surveys. The definition of positive hormone receptor status was standardized using the following criteria: ≥ 10% cells stained, any “plus-system description”, ≥ 20 fmol/mg, an Allred score of ≥ 3, immunoreactive score (IRS) ≥ 2, or an H-score ≥ 10. Among the WHI women, centrally trained, locally based physician adjudicators verified BC diagnoses by medical record and pathology report review, and positive hormone receptor status was defined as ≥ 10% cells stained [15].

### Absolute risk modeling

Using the EPIC data, we fitted cause-specific piecewise-constant hazards models [16] for ER+ and ER- tumors separately (hereinafter referred to as Model_ER+_ and Model_ER-_). The cutoffs were placed at 45, 50, 55, 60, 65, 70, and 75 years of age. Whether a risk association is heterogeneous by ER status was examined using the likelihood ratio test [17].

Tumors with unknown ER status, primary cancers at other sites, and deaths from non-cancer causes were modelled as competing events to ER+ tumors and ER- tumors. A Gompertz model with age as the time scale was fitted for all these competing events combined. In addition, ER+ and ER- tumors were considered mutually competing.

To evaluate the improvement in risk prediction by modeling the heterogeneous risk associations, an omnibus model was also fitted following the same methodology described above, treating ER+ and ER- tumors as one single disease outcome.

### Model validation

First, we validated our ER-specific models internally by fivefold cross-validation [18] and then externally using WHI data. For external validation using the WHI data, we combined the model coefficients derived from the EPIC women and the ER-specific baseline hazards of the WHI women to project 5-year ER-specific absolute risks. We calculated *C*-statistics to assess discriminatory accuracy and the ratio of expected-to-observed number of tumors occurring in the first 5 years (E/O) to assess overall calibration. In the WHI women, the 5-year absolute risk of developing BC was projected using Model_Gail_, enabling us to compare the performance of our model with that of Model_Gail_.

We performed decision curve analysis in the WHI women to compare the clinical applicability of Model_ER+_ and Model_Gail_ for identification of women for chemoprevention.

Let B denote the benefit of receiving chemoprevention for an individual who would develop BC, H the harm of receiving chemoprevention for an individual who would never develop BC, and p_i_ indicates an individual risk. The rationale of decision curve analysis is that positive net benefits is guaranteed at the population level if chemoprevention only covers individuals with risk projections p_i_ above the risk threshold p_t_, where:

p_t_ × B = (1 − p_t_) × H [19, 20].

Given the fact that quantities of B and H of chemoprevention remain unknown, net benefits are calculated through all the possible risk thresholds between two extremes, i.e. zero and the maximal risk estimate, representing a treat-all strategy and a treat-none strategy, respectively. The clinical applicability of a risk prediction model is indicated by how much the model’s net benefit curve is above the treat-all and treat-none strategies, i.e. the area formed by the model’s net benefit curve and the two extreme strategies.

## Results

### Cohort description

Country-specific distributions of the risk factors among the EPIC women are shown in Table 6 in Appendix. Distributions of the same risk factors among the WHI women are shown in Table 7 in Appendix. During an average follow-up period of 14.7 years, 12,067 BC cases (7210 ER+ tumors, 1598 ER- tumors, and 3259 tumors with unknown ER status), 16,929 primary cancers at other sites, and 6548 deaths from non-cancer causes were ascertained among the EPIC women, as reported in Table 1.

**Table 1:** Distribution of incident breast cancer (BC) by country, estrogen receptor (ER) status, and baseline menopausal status among the women from the European Prospective Investigation into Cancer and Nutrition (EPIC) and Women’s Health Initiative (WHI) studies

	Number	Age at recruitment (years)	Years of follow-up	Incident BC	Crude incidence rate (/10^5^ person-years)	Premenopausal				Postmenopausal			
						ER+	ER-	Indefinite	BC	ER+	ER-	Indefinite	BC
EPIC study													
France	68,707	51.5	14.7	3386	382	1232	323	221	1776	1150	254	206	1610
Italy	27,851	52.0	15.0	1135	287	401	88	92	581	402	70	82	554
Spain	20,298	50.2	16.7	556	171	164	43	92	299	148	38	71	257
UK	35,349	52.3	16.1	1602	300	328	76	267	671	475	65	391	931
Netherlands	22,601	54.8	15.0	975	305	206	35	143	384	333	55	203	591
Greece	11,337	55.6	11.7	201	158	19	6	44	69	35	3	94	132
Germany	22,085	52.6	11.6	743	322	198	52	49	299	308	73	63	444
Sweden	9142	50.5	16.4	370	247	143	38	38	219	87	27	37	151
Denmark	29,309	56.3	16.4	1887	428	257	61	100	418	921	207	341	1469
Norway	34,651	48.0	14.1	1212	263	245	54	480	779	158	30	245	433
Total	281,330	52.1	14.7	12,067	312	3193	776	1526	5495	4017	822	1733	6572
WHI study	82,319	63.2	8.2	2951	457	–	–	–	–	2276	421	254	2951

### The ER-specific absolute risk models

Among the risk factors with identical associations by ER status (Table 2), being postmenopausal compared with premenopausal at recruitment was associated with a reduced tumor risk after controlling for age (hazard ratio (HR) = 0.66, 95% confidence interval (CI) = 0.60 to 0.74). For postmenopausal women, a statistically significant and monotonically increasing tumor risk was observed with older age at menopause compared with reaching menopause before the age of 45 years (*p*_trend_ < 0.001). No statistically significant association was observed for breastfeeding and breast cancer risk among parous women. Later age at menarche (≥ 15 vs ≤ 11 years of age) was statistically significantly associated with decreased tumor risk (HR = 0.85, 95% CI = 0.79 to 0.92). Duration of HRT was statistically significantly associated with increased breast cancer risk (*p*_trend_ < 0.001). BMI was associated with breast cancer and exhibited a statistically significant interaction with menopausal status: for postmenopausal women, HRs (95% CIs) for the BMI categories in ascending order were 1.11 (1.04 to 1.18), 1.21 (1.10 to 1.34), and 1.30 (1.11 to 1.53), respectively. For alcohol intake, exceeding one drink per day, compared with nondrinking, was statistically significantly associated with an increased breast cancer risk.

**Table 2:** Risk associations for ER+ and ER- tumors, the EPIC study and the WHI study^a^

Risk factors	EPIC study		WHI study	
	ER+, *n* = 7210 HR (95% CI)	ER-, *n* = 1598 HR (95% CI)	ER+, *n* = 2276 HR (95% CI)	ER-, *n* = 421 HR (95% CI)
Menopausal status:				
postmenopausal^b^ vs premenopausal	0.66 (0.60–0.74)		–	
Age at menopause, years:				
45.1–50.0 vs ≤ 45.0	1.16 (1.06–1.28)		1.16 (1.05–1.29)	
50.1–55.0 vs ≤ 45.0	1.25 (1.13–1.38)		1.41 (1.27–1.56)	
> 55.0 vs ≤ 45.0	1.41 (1.21–1.63)^e^		1.40 (1.20–1.62)	
Breastfeeding, months:				
0.1–6 vs 0	1.01 (0.95–1.08)		1.04 (0.94–1.14)	
6.1–12 vs 0	0.96 (0.88–1.04)		1.04 (0.91–1.18)	
> 12 vs 0	1.01 (0.93–1.11)		1.07 (0.95–1.20)	
Age at menarche, years:				
12 vs ≤ 11	1.06 (0.98–1.14)		0.89 (0.80–0.99)	
13 vs ≤ 11	1.00 (0.93–1.07)		0.82 (0.74–0.91)	
14 vs ≤ 11	0.97 (0.91–1.05)		0.86 (0.75–0.98)	
≥ 15 vs ≤ 11	0.85 (0.79–0.92)		0.78 (0.67–0.91)	
HRT use, years:				
0.1–1.0 vs 0	1.17 (1.09–1.26)		1.01 (0.86–1.19)	
1.1–2.0 vs 0	1.27 (1.15–1.40)		1.17 (0.97–1.40)	
2.1–3.0 vs 0	1.39 (1.24–1.56)		1.37 (1.13–1.65)	
> 3.0 vs 0	1.55 (1.44–1.66)^e^		1.53 (1.39–1.67)^e^	
BMI, kg/m^2^:				
25.0–29.9 vs < 25.0	0.99 (0.92–1.07)		1.02 (0.93–1.12)	
30.0–34.9 vs < 25.0	0.97 (0.85–1.10)		1.14 (1.02–1.28)	
≥ 35.0 vs < 25.0	1.12 (0.92–1.36)		1.23 (1.07–1.41)	
BMI* menopause^c^:				
1 vs 0	1.11 (1.01–1.23)		–	
2 vs 0	1.26 (1.07–1.47)		–	
3 vs 0	1.17 (0.91–1.50)		–	
Alcohol intake, drinks per day:				
< 1.0 vs 0	1.00 (0.94–1.07)		1.08 (0.91–1.29)	
1.0–1.9 vs 0	1.14 (1.05–1.24)		1.20 (0.98–1.47)	
≥ 2.0 vs 0	1.22 (1.12–1.33)		1.26 (1.01–1.59)	
FTP^d^:				
Yes vs no	0.81 (0.71–0.91)	0.97 (0.76–1.24)	0.85 (0.67–1.08)	0.65 (0.37–1.14)
Number of FTP:				
2 vs 1	0.99 (0.92–1.06)	1.05 (0.90–1.22)	1.14 (0.97–1.34)	1.15 (0.78–1.70)
≥ 3 vs 1	0.87 (0.80–0.95)	0.95 (0.81–1.13)	0.99 (0.84–1.17)	0.96 (0.65–1.41)
Age at 1st FTP, years:				
20.1–25.0 vs ≤ 20.0	1.05 (0.97–1.14)	1.04 (0.89–1.23)	1.03 (0.89–1.20)	1.37 (0.97–1.95)
25.1–30.0 vs ≤ 20.0	1.20 (1.10–1.31)	0.93 (0.78–1.12)	1.14 (0.97–1.33)	1.34 (0.92–1.96)
30.1–35.0 vs ≤ 20.0	1.32 (1.18–1.48)	0.96 (0.75–1.23)	1.59 (1.31–1.94)	1.01 (0.58–1.75)
> 3.05 vs ≤ 20.0	1.46 (1.24–1.73)^e^	0.91 (0.59–1.38)	1.56 (1.16–2.09)	1.10 (0.48–2.53)
Height, per 10-cm increment	1.19 (1.15–1.24)	1.06 (0.98–1.16)	1.14 (1.06–1.22)	1.04 (0.89–1.22)

*BMI* body mass index, *CI* confidence interval, *EPIC* European Prospective Investigation into Cancer and Nutrition, *ER* estrogen receptor, *FTP* full-term pregnancy, *HR* hazard ratio, *HRT* hormone replacement therapy, *WHI* Women’s Health Initiative

^a^Heterogeneous risk associations among the EPIC women were examined using the likelihood ratio test. The resulting heterogeneous risk factor profiles were applied to the WHI women

^b^Age at menopause ≤ 45 years

^c^0: premenopausal or postmenopausal and BMI < 25 kg/m^2^; 1: postmenopausal and BMI 25.0–29.9 kg/m^2^; 2: postmenopausal and BMI 30.0–34.9 kg/m^2^; 3: postmenopausal and BMI ≥ 35 kg/m^2^. Among postmenopausal women, the HRs (95% CIs) for BMI from low to high categories were 1.11 (1.04–1.18), 1.21 (1.10–1.34), and 1.30 (1.11–1.53)

^d^The number of FTP = 1 and age at first FTP ≤ 20 years.

^e^*p*_trend_ < 0.001

Tests for heterogeneity showed differential risk associations for FTP, number of FTPs, age at first FTP, body height, and country by ER status (Table 2 and Table 8 in Appendix). Parity (one single FTP, age at FTP ≤ 20 years) compared with nulliparity was associated with a statistically significant reduction in ER+ tumor risk (HR = 0.81, 95% CI = 0.71 to 0.91). Among parous women, having three or more FTPs was associated with a further risk reduction for ER+ tumors compared with one single FTP (HR = 0.87, 95% CI = 0.80 to 0.95), and delayed age at first FTP was associated with increased ER+ tumor risk (*p*_trend_ < 0.001). In addition, every 10-cm increment in body height was associated with a 19% increase in ER+ tumor risk (95% CI = 1.15 to 1.24). None of these factors, however, was statistically significantly associated with ER- tumor risk. Table 8 in Appendix shows the coefficients for different countries by ER status. Based on the same heterogeneous risk factor profiles, we also estimated the risk associations using the WHI data (Table 2), which were largely comparable to those from the EPIC study, with the exception of age at menarche, and especially for ER- tumors, FTP, number of FTP, and age at first FTP.

### Model validation

Table 3 shows the predictive performance of the ER-specific models (*C*-statistic and E/O) corrected by the fivefold cross-validation. Model_ER+_, Model_ER-_ and the omnibus model shared a *C*-statistic of 0.68. Elimination of the country effect reduced the *C*-statistic notably to 0.64 for Model_ER+_, 0.59 for Model_ER-_, and 0.63 for the omnibus model. A minor difference in *C*-statistic was observed between premenopausal and postmenopausal women. The omnibus model exhibited a higher *C*-statistic for ER+ than for ER- tumors (0.64 vs 0.59). Model_ER+_ significantly overestimated the 5-year tumor risk by 10% (E/O = 1.10, 95% CI = 1.05 to 1.14), particularly among premenopausal women (13%). Model_ER-_ non-significantly underestimated the risk (E/O = 0.96, 95% CI = 0.88 to 1.05) overall and by menopausal status.

**Table 3:** Internal validation of the estrogen receptor (ER)-specific risk prediction models (Model_FR+_ and Model_FR-_) by fivefold cross-validation, overall and by age, in the women from the European Prospective Investigation into Cancer and Nutrition (EPIC) study

	Model_ER+_	Model_ER-_	Omnibus model
*C*-statistic (95% CI)			
Before eliminating country effect	0.68 (0.65–0.70)	0.68 (0.64–0.72)	0.68 (0.66–0.70)
After eliminating country effect			
Overall	0.64 (0.61–0.67)	0.59 (0.54–0.64)	0.63 (0.60–0.65)
By menopausal status			
Premenopausal	0.64 (0.59–0.68)	0.58 (0.51–0.66)	0.62 (0.59–0.66)
Postmenopausal	0.62 (0.59–0.66)	0.60 (0.52–0.67)	0.62 (0.59–0.65)
By ER status			
ER+	–	–	0.64 (0.62–0.67)
ER−	–	–	0.59 (0.53–0.64)
Ratio of observed–expected (95% CI)			
Overall	1.10 (1.05–1.14)	0.96 (0.88–1.05)	1.07 (1.03–1.11)
By menopausal status			
Premenopausal	1.13 (1.06–1.20)	0.97 (0.85–1.10)	1.09 (1.02–1.15)
Postmenopausal	1.07 (1.02–1.13)	0.96 (0.84–1.08)	1.06 (1.00–1.11)

**Fig. 1:**
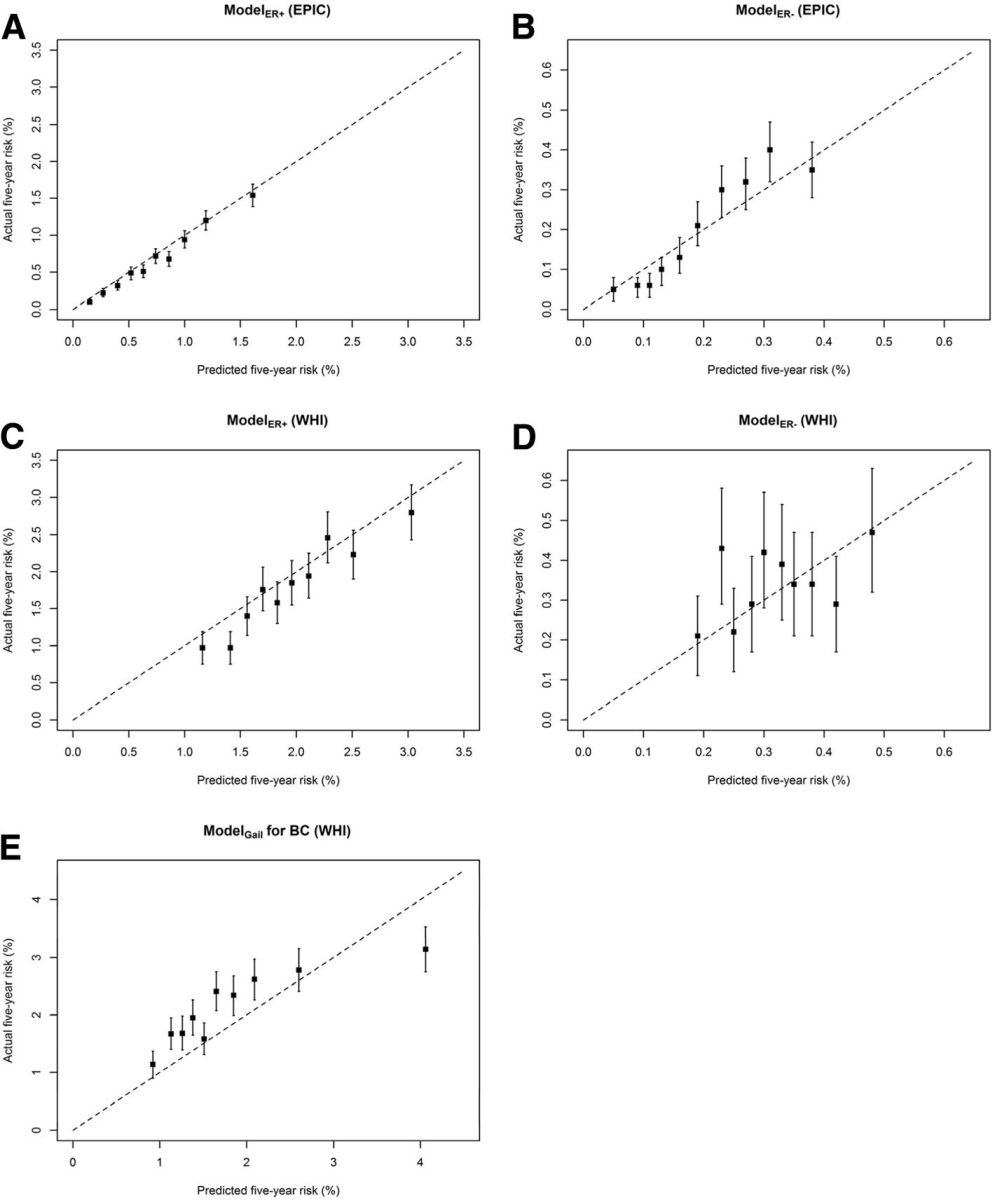
Calibration of the risk prediction model of ER-positive tumors (Model_ER+_), risk prediction model of ER-negative tumors (Model_ER-_), and Gail risk prediction model (Model_Gail_) by risk deciles. **a** Model_ER+_ in women from the European Prospective Investigation into Cancer and Nutrition (EPIC); **b** Model_ER-_ in the EPIC women; **c** Model_ER+_ in the women from the Women’s Health Initiative (WHI); **d** Model_ER-_ in the WHI women; **e** Model_Gail_ in the WHI women

External validation with the WHI data resulted in a *C*-statistic of 0.59 (95% CI = 0.58 to 0.60) for Model_ER+_ and 0.53 (95% CI = 0.50 to 0.57) for Model_ER-_ (Table 4). Model_Gail_ yielded an overall *C*-statistic of 0.57 (95% CI = 0.56 to 0.59) with a markedly lower *C*-statistic of 0.53 (95% CI = 0.50 to 0.57) for ER- tumors. Regarding calibration, an overestimation was observed for ER+ tumors (*E/O* = 1.09, 95% CI = 1.03 to 1.14) whereas a statistically non-significant underestimation was observed for ER- tumors (E/O = 0.94, 95% CI = 0.82 to 1.06). Model_Gail_ underestimated the overall BC risk by 22% (E/O = 0.78, 95% CI = 0.73 to 0.82). Among the EPIC women, the overestimation of ER+ tumor risk occurred largely in low-risk individuals (Fig. 1a); for ER- tumor risk, overestimation was observed mainly among low-risk individuals whereas underestimation was observed mainly among high-risk individuals (Fig. 1b). Among WHI women, the overestimation by Model_ER+_ and the underestimation by Model_Gail_ were largely systematic (Fig. 1c and e). The statistically non-significant underestimation by Model_ER-_ in the WHI women showed no clear pattern (Fig. 1d).

**Table 4:** External validation of the estrogen receptor (ER)-specific risk prediction models and the Gail model in women from the Women’s Health Initiative (WHI) study

	ER-specific risk prediction models		Model_Gail_
	Model_ER+_	Model_ER−_	
*C*-statistic (95% CI)			
Overall	0.59 (0.58–0.60)	0.53 (0.50–0.57)	0.57 (0.56–0.59)
By ER status			
ER+	–	–	0.58 (0.57–0.60)
ER−	–	–	0.53 (0.50–0.57)
Ratio of observed–expected (95% CI)	1.09 (1.03–1.14)	0.94 (0.82–1.06)	0.78 (0.73–0.82)

**Fig. 2:**
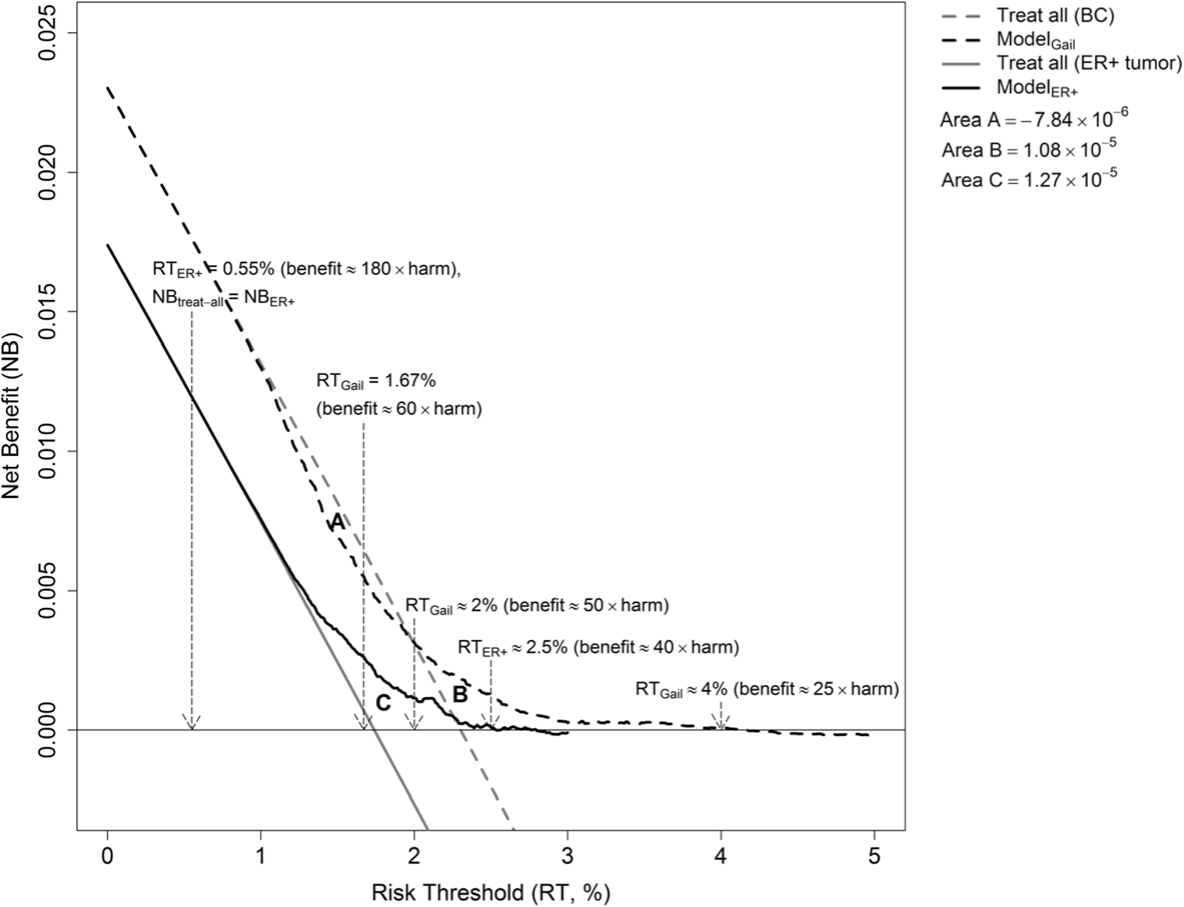
Net benefit curves for the risk prediction model of ER-positive tumors (Model_ER+_) (black solid line) and the Gail risk prediction model (Model_Gail_) (black broken line) applied to women from the Women’s Health Initiative study. Corresponding curves for the treat-all strategy are represented in gray (solid line for all breast cancer cases, broken line for ER+ tumors only). Area A = − 7.84 × 10^-6^; Area B = 1.08 × 10^-5^; Area C = 1.27 × 10^-5^

Figure 2 shows the net benefit curves of Model_ER+_ and Model_Gail_. The net benefit curves of the two models started to diverge from the treat-all strategies at the risk threshold of 0.55%, which was roughly the minimal risk projected by both models. Model_ER+_ would yield higher net benefits than both the treat-all strategy and the treat-none strategy (denoted by the x-axis at y = 0) if the risk threshold lay between 0.55% and 2.5%, corresponding to an assumption that the benefit of chemoprevention was 180 to 40 times the harm. In contrast, Model_Gail_ would yield lower net benefits than the treat-all strategy if a risk threshold below 2% were selected, including 1.67%, the currently adopted risk threshold for chemoprevention in the USA, and would yield negative net benefits if a risk threshold above 4% (i.e. benefit ≈ 25 × harm) were selected. The clinical applicability of Model_Gaili_, as indicated by the sum of **Area A** and **Area B** shown in Fig. 2, was 3.0 × 10^− 6^. The clinical applicability of Model_ER+_ was 12.7 × 10^− 6^ (**Area C**).

## Discussion

The heterogeneous risk associations in our ER-specific risk prediction models are consistent with the established knowledge that FTP, number of FTPs, and delayed childbirth are associated with ER+ tumors but not with ER- tumors [6–8]. Our study also confirms a largescale meta-analysis of epidemiological data showing that BC risk increases with prolonged duration of HRT use [21]. Data from the WHI randomized trial showed a statistically significant increase in the incidence and mortality of invasive BC in the estrogen-plus-progestin arm compared with the placebo arm [22, 23], whereas estrogen alone decreased BC incidence and mortality among postmenopausal women with prior hysterectomy [24, 25]. Stronger positive associations for estrogen plus progestin than for estrogen alone were reported for BC [26, 27]. In the present study, we could not separate estrogen alone and estrogen plus progestin due to unknown HRT compounds among former users in EPIC. Among current HRT users at baseline, use of estrogen plus progestin was more common in EPIC than in the WHI cohort (76% vs 44%, respectively). However, similar associations between the duration of lifetime HRT use and BC risk were observed in both the EPIC and the WHI study.

In ER-specific risk models, statistically significant and homogeneous risk associations were fitted for age at menopause and age at menarche, in line with a pooled analysis of previous investigations where nearly identical effects were observed for ER+ tumors and ER- tumors [28]. The present study demonstrated a null association between breastfeeding and BC risk, inconsistent with previous investigations where inverse associations were reported [6, 8, 29]. We note that most previous studies were case-control studies, which were subject to recall bias. In fact, the inverse association disappeared in some cohort studies [30, 31]. In a more recent pooled analysis, breastfeeding was not associated with ER+ and/or PR+ tumors but was inversely associated with ER-/PR- tumors [32].

In a pooled analysis of prospective cohort data, every 10-cm increment in body height was statistically significantly associated with ER+ tumor risk (HR = 1.18) but had null association with ER- tumor risk [33], supporting the way we modeled body height in the present study.

Prediction of ER+ tumor risk might be practically more useful than prediction of overall BC risk [3]. The reason for this is twofold. First, projecting subtype-specific risks allows for accurate estimation of the risk associations of factors that are etiologically heterogeneous and as a result might increase the discriminatory power. Second, since currently used chemoprevention only reduces the risk of ER+ tumors [34], there is a need for a model that can specifically predict the risk of developing ER+ tumors.

The discriminatory accuracy of Model_ER+_ in internal validation performed no better than most of the current omnibus models using questionnaire-derived data, suggesting limited improvement in discrimination after accounting for etiological heterogeneity. This was not surprising given that ER+ tumors are the dominant subtype and the omnibus model shared nearly equivalent parameters (data not shown) with Model_ER+_ in the present study. According to the only study so far that has modeled ER-specific risks, the discriminatory power of the ER+/PR+ model was moderately higher than that of the ER-/PR- model (0.64 vs 0.61) [10]. In that study, risk factors with heterogeneous associations included age, menopausal status, BMI, age at first birth, and past use of postmenopausal HRT, and its subtype-specific models were based on a relatively small number of tumors (1281 ER+/PR+ tumors, 417 ER-/PR- tumors). Notably, in that study there was no correction for potential overfitting by either internal or external approaches.

When externally validated in the WHI cohort, Model_ER+_ exhibited moderate discriminatory accuracy comparable to that of Model_Gail_. Women in the USA with 5-year BC risk of 1.67% or higher, projected by Model_Gail_, are considered potentially eligible for chemoprevention [35]. This risk threshold would lead to coverage of 36,265 (44.0%) women in our WHI validation population, of whom 1239 were subsequently diagnosed with ER+ tumors and 194 with ER- tumors. According to Model_ER+_, a risk threshold of 1.97% would cover the same number of women with 16 more prospective ER+ tumors and 2 fewer prospective ER- tumors.

The decision curve analysis provided some interesting insight into the clinical applicability of Model_ER+_ and Model_Gail_. As indicated by the net benefit curves, Model_ER+_ would demonstrate no advantage over the treat-all strategy if the benefit-to-harm ratio of chemoprevention were higher than 180, equivalent to any risk threshold below the minimal risk projection (≈ 0.55%), while such a boundary benefit-to-harm ratio was 50 for Model_Gail_. Interestingly, the treat-all strategy would even outperform Model_Gail_ when the risk threshold was situated at 1.67%. In contrast to Model_Gail_, Model_ER+_ had a wider threshold range where higher net benefits could be obtained by a model-based decision-making than by either the treat-all or the treat-none strategy. Considering the unknown benefit and harm associated with chemoprevention, Model_ER+_ thus has broader applicability than Model_Gail_, as indicated by the areas formed by the two models’ net benefit curves and the two extreme strategies. As shown in Fig. 2, the lowest benefit-to-harm ratio for chemoprevention against BC to produce a positive net benefit is 25, whereas such a benefit-to-harm ratio for chemoprevention against ER+ tumors is 40, suggesting that chemoprevention against ER+ tumors might be 1.6 times (40/25) more efficient than chemoprevention against all types of BC.

Among both the EPIC women and the WHI women, Model_ER+_ overestimated the 5-year risk by about 10%, possibly due to potential misspecifications of our models, such as imperfect fit of the baseline hazard functions (the baseline hazard estimates are given in Table 9 in the Appendix). More importantly, this overestimation was systematic rather than in an overfitting pattern, i.e. underestimation occurs in low-risk individuals and overestimation occurs in high-risk individuals [36].

We derived ER-specific models from a large prospective cohort and validated them in another large independent cohort for external validation. This is a strong approach to robust parameterization and assessment of model performance. However, some limitations characterize the present study. Our models did not include some established risk factors such as family history of BC (FHBC) and previous breast biopsy, as these variables were not available in the EPIC study. A complete-case analysis of EPIC women with known FHBC (*n* = 138,257, 49% of the sample) showed positive homogenous associations between FHBC and tumor subtypes (HR_ER+_ = 1.64, 95% CI = 1.49 to 1.81; HR_ER-_ = 1.50, 95% CI = 1.23 to 1.91; *p*_heterogeneity_ = 0.57), suggesting that inclusion of this factor would increase the predictive power of the model, though not differentially across the hormonal receptor status of the tumors. Another limitation of our study was the underestimation of baseline hazards due to EPIC tumors with unknown ER status, which accounted for about 25% of BC diagnoses. Under the assumption of ER-status data missing at random, parameter estimates are expected to be unbiased, a necessary requisite to carry out proper external validation, whereas the underestimated baseline hazard would be replaced with the actual baseline hazard function of the test population.

## Conclusions

In summary, we found that modeling heterogeneous risk associations of epidemiological factors yields little improvement in BC risk prediction. Nevertheless, compared with the current omnibus models, a model specifically predictive of ER+ tumor risk could be more applicable in risk assessment for chemoprevention.

## Appendix

### Description of the Gail model

The Gail model, also known as the Breast Cancer Risk Assessment Tool, has been adopted to estimate the 5-year absolute risk of developing invasive breast cancer among women aged 35 years or older. Women with a 5-year absolute risk of 1.67% or higher as projected by the Gail model are regarded as eligible for chemoprevention by tamoxifen. The Gail model includes the following predictor variables: age, ethnicity, age at menarche, age at first live birth, number of first-degree relatives with breast cancer, number of previous breast biopsies, and history of atypical hyperplasia. The relative risks of these risk factors were estimated from a case-control study within the Breast Cancer Detection Demonstration Project (BCDDP). The baseline age-specific hazard rates were also calculated from the BCDDP as the observed age-specific hazard rates times 1 minus the population attributable fraction [1]. Five-year breast cancer risk projection in the Women’s Health Initiative study using the Gail model has been detailed elsewhere [3].

**Table 5:** Coding for predictor variables

Predictor variable	Coding
Menopausal status	0: premenopausal 1: postmenopausal
Age at menopause	0: premenopausal or ≤ 45 years 1: 45.1–50.0 years 2: 50.1–55.0 years 3: > 55.0 years
Breastfeeding	0: parous women who never breastfed and nulliparous women 1: 0.1–6 months 2: 6.1–12 months 3: > 12 months
Age at menarche	0: ≤ 11 years 1: 12 years 2: 13 years 3: 14 years 4: ≥ 15 years
Hormone replacement therapy (HRT) use	0: never used HRT 1: 0.1–1 year 2: 1.1–2 years 3: 2.1–3 years 4: > 3 years
Full-term pregnancy (FTP)	0: nulliparous 1: parous
Number of FTPs	0: nulliparous or 1 FTP 1: 2 FTPs 2: ≥ 3 FTPs
Age a first FTP	0: nulliparous or ≤ 20 years 1: 20.1–25.0 years 2: 25.1–30.0 years 3: 30.1–35.0 years 4: > 35.0 years
Body mass index	0: < 25.0 kg/m^2^ 1: 25–29.9 kg/m^2^ 2: 30–34.9 kg/m^2^ 3: > 35.0 kg/m^2^
Body height per 10-cm increment	Body height in centimeters divided by 10, continuous variable
Alcohol intake	0: non-drinker 1: < 1 drink/day 2: 1.0–1.9 drinks/day 3: ≥ 2 drinks/day

**Table 6:** Distributions of the risk factors among the EPIC women by country

	France	Italy	Spain	UK	Netherlands	Greece	Germany	Sweden	Denmark	Norway	Total
	*n* = 68,707	*n* = 27,851	*n* = 20,298	*n* = 35,349	*n* =22,601	*n* = 11337	*n* = 22,085	*n* = 9142	*n* = 29,309	*n* = 3,4651	*n* = 281,330
Menopausal status:											
Premenopausal	54.0	49.4	54.0	46.4	39.1	35.0	48.6	61.7	22.6	69.5	48.5
Postmenopausal	46.0	50.6	46.0	53.6	60.9	65.0	51.4	38.3	77.4	30.5	51.5
Age at menopause, years:											
≤ 45.0	9.7	19.1	26.1	19.5	22.6	30.7	18.3	10.6	16.8	10.4	17.2
45.1–50.0	20.4	43.9	39.1	29.2	35.6	36.0	33.6	32.7	33.5	22.5	30.9
50.1–55.0	21.7	28.3	28.0	22.2	29.2	26.2	26.2	18.2	27.5	9.9	24.1
> 55.0	3.5	2.5	2.8	3.2	3.2	2.5	3.0	3.1	3.9	0.0	3.0
Missing^a^	44.7	6.2	4.0	26	9.4	4.6	20	35.4	18.3	57.2	24.8
FTP:											
0 (no)	9.0	12.1	9.8	15.5	13.2	8.6	13.5	4.5	11.6	8.5	10.9
1 (yes)	83.8	86.6	89.4	80.8	85.5	90.8	86.2	57.4	88.0	91.5	85.2
Missing	7.2	1.3	0.8	3.7	1.3	0.6	0.3	38.1	0.4	0.0	3.9
Number of FTP:											
1	18.4	24.9	9.1	15.6	6.9	11.3	29.6	13.8	17.7	13.3	16.8
2	49.4	49.9	37.4	49.9	31.3	55.2	49.2	47.4	51.4	49.2	47.5
≥ 3	32.2	25.2	53.5	34.3	33.3	33.4	21.2	37.9	30.9	37.5	33.3
Missing^b^	0.0	0.0	0.0	0.2	28.5	0.1	0.0	0.9	0.0	0.0	2.4
Age at 1^st^ FTP, years:											
≤ 20	9.8	8.7	10.4	11.8	10.0	23.3	20.2	17.1	23.6	21.5	14.6
20.1–25.0	53.1	43.6	52.2	42.2	45.1	41.5	47.3	45.5	46.4	45.9	47.3
25.1–30.0	27.8	34.6	30.3	31.8	34.8	25.0	23.1	25.9	22.5	23.6	28.1
31.1–35.0	7.0	10.2	5.5	10.1	7.9	6.8	7.1	6.2	5.0	6.8	7.4
> 35.0	2.0	2.7	1.5	3.6	2.2	2.3	2.2	1.6	1.3	2.2	2.2
Missing^b^	0.3	0.2	0.1	0.5	0.0	1.1	0.1	3.7	1.2	0.0	0.4
Breastfeeding, months:											
0	27.7	16.8	11.2	15.8	13.6	11.1	16.9	0	6.9	5.7	15.5
0.1–6.0	47.2	39.6	33.0	40.1	34.6	31.2	59.6	0	43.8	26.5	39.7
6.1–12.0	14.0	23.4	21.1	16.2	13.1	16.2	13.8	0	26.1	24.0	18.0
> 12.0	5.4	20.0	34.4	22.6	9.8	40.3	9.2	0	21.0	42.2	19.7
Missing^b^	5.7	0.2	0.3	5.3	28.9	1.2	0.5	100	2.2	1.6	7.1
Age at menarche, years:											
≤ 11	17.0	23.2	18.1	19.2	10.7	9.7	10.8	6.1	8.9	8.5	14.6
12	24.6	25.0	19.6	18.1	19.9	22.5	21.9	12.0	13.6	19.8	20.7
13	26.0	24.3	23.8	23.9	23.9	25.9	25.5	17.9	22.4	28.7	25.0
14	20.6	17.6	22.4	19.0	21.8	20.1	23.0	15.1	25.2	24.4	21.1
≥ 15	11.2	8.5	16.1	14.3	21.6	20.7	18.6	11.4	26.2	17.1	15.6
Missing	0.6	1.4	0.0	5.5	2.1	1.1	0.2	37.5	3.7	1.5	3.0
HRT:											
1 (yes)	31.0	17.2	11.3	31.7	22.5	5.6	38.5	14.0	43.9	31	28.0
0 (no)	68.3	81.1	85.7	64.9	74.5	93.8	25.7	45.4	54.2	69	66.4
Missing	0.7	1.7	3.0	3.4	3.0	0.6	35.8	40.6	1.9	0	5.6
Duration of HRT, years:											
≤ 1.0	26.4	55.2	62.0	30.0	38.0	62.0	21.6	19.2	27.9	18.3	29.2
1.1–2.0	21.0	11.7	17.1	14.1	13.8	13.5	11.3	12.3	10.2	13.3	14.8
2.1–3.0	14.0	8.3	6.6	10.8	9.3	5.9	10.3	7.6	8.2	10.0	10.6
> 3.0	38.6	17.1	12.0	37.9	31.8	16.8	50.7	20.5	52.7	30.1	38.0
Missing^c^	0.0	7.7	2.3	7.2	7.1	1.8	6.1	40.4	1.0	28.3	7.4
Height, cm, median	161	158	156	162	164	156	163	164	164	167	162
BMI, kg/m^2^:											
< 25.0	77.2	46.9	21.1	58.2	50.6	20.9	47.4	57.3	51.1	64.2	56.0
2.05–29.9	18.5	37.5	43.7	30.3	36.4	38.4	34.6	31.5	34.6	27.7	30.4
30.0–34.9	3.5	11.8	25.3	8.5	10.1	27.8	13.1	8.3	10.6	6.4	10.1
≥ 35.0	0.8	3.8	9.9	3.0	2.9	12.9	4.9	2.9	3.7	1.7	3.5
Alcohol intake, drinks/day:											
0	14.0	22.4	53.3	7.4	17.1	35.3	4.6	16.4	2.7	21.0	17.0
< 1.0	54.4	46.6	34.2	69.5	56.4	57.2	70.8	82.9	58.2	77.5	59.8
1.0–1.9	18.0	16.6	8.5	10.9	14.6	5.4	14.9	0.7	20.8	1.5	13.0
≥ 2.0	13.6	13.1	4.0	6.4	11.4	1.6	9.6	0	18.2	0	9.3
Missing	0	1.3	0	5.8	0.5	0.5	0.1	0	0.1	0	0.9

Values are percentages unless otherwise indicated

*BMI* body mass index, *EPIC* European Prospective Investigation into Cancer and Nutrition, *FTP* full-term pregnancy, *HRT* hormone replacement therapy

^a^Among postmenopausal women

b‡Among parous women (FTP = 1)

^c^Among HRT users

**Table 7:** Distributions of the risk factors among the WHI women by ER status

	Tumor-free	ER+	ER-	Indefinite
	*n* = 79,368	*n* = 2276	*n* = 421	*n* = 254
Age at menopause, years:				
≤ 45	26.8	22.8	25.6	25.6
45.1–50.0	34.4	31.9	38.0	35.8
50.1–55.0	30.3	34.9	30.0	28.7
> 55.0	8.5	10.4	6.4	9.9
Breastfeeding, months:				
0	47.9	45.6	48.7	50.8
0.1–6.0	25.8	26.5	26.1	26.0
6.1–12.0	11.4	12.5	8.8	11.0
> 12.0	14.9	15.4	16.4	12.2
Age at menarche, years:				
≤ 11	21.5	24.0	23.0	22.4
12	26.2	26.2	28.3	23.6
13	30.2	28.5	28.3	33.1
14	13.1	13.3	11.9	13.0
≥ 15	9.0	8.0	8.5	7.9
HRT use, years:				
0	33.3	27.0	30.4	31.5
0.1–1.0	8.5	6.6	8.3	8.7
1.1–2.0	5.5	5.3	4.5	6.7
2.1–3.0	4.4	4.8	4.3	4.3
> 3.0	48.3	56.3	52.5	48.8
BMI, kg/m^2^:				
< 25.0	38.8	37.8	42.3	29.5
25.0–29.9	35.2	35.2	30.1	33.9
30–34.9	16.7	17.1	18.8	21.6
≥ 35.0	9.3	9.9	8.8	15.0
Alcohol intake, drinks/day:				
0	5.6	5.1	4.3	5.9
< 1.0	77.7	75.4	8.0	76.0
1.0–1.9	11.2	12.7	12.4	13.4
≥ 2.0	5.4	6.8	0.3	4.7
FTP:				
0 (no)	10.3	10.0	11.2	7.1
1 (yes)	89.7	90.0	88.8	92.9
Number of FTP:				
1	9.5	9.7	8.8	12.3
2	30.2	33.2	34.5	27.5
≥ 3	60.3	57.1	56.7	60.2
Age at 1^st^ FTP, years:				
≤ 20.0	13.0	11.0	9.9	16.5
20.1–25.0	49.4	45.6	52.4	44.9
25.1–30.0	28.5	30.0	30.2	28.8
30.1–35.0	7.0	10.3	5.6	8.5
> 35.0	2.1	3.1	1.8	1.3
Height, cm, mean	162.2	162.6	162.4	162.9

Values are percentages unless otherwise indicated

*BMI* body mass index, *ER* estrogen receptor, *FTP* full-term pregnancy, *HRT* hormone replacement therapy, *WHI* Women’s Health Initiative

**Table 8:** Coefficients for different countries by ER status in the women from the EPIC study

	Model_ER+_		Model_ER−_	
	Coefficient	*P*	Coefficient	*P*
France	0	–	0	–
Italy	−0.186	< 0.001	−0.397	< 0.001
Spain	−0.791	< 0.001	−0.806	< 0.001
UK	−0.582	< 0.001	−0.842	< 0.001
Netherlands	−0.513	< 0.001	−0.762	< 0.001
Greece	−1.610	< 0.001	−2.023	< 0.001
Germany	−0.214	< 0.001	−0.211	0.035
Sweden	−0.473	< 0.001	−0.299	0.024
Denmark	−0.179	< 0.001	−0.082	0.302
Norway	−1.006	< 0.001	−1.244	< 0.001

*Model*_*ER+*_ risk prediction model of ER-positive tumors, Model_ER-_ risk prediction model of ER-negative tumors, *EPIC* European Prospective Investigation into Cancer and Nutrition, *ER* estrogen receptor

**Table 9:** Baseline piecewise constant hazard estimates for postmenopausal ER+ tumors and ER- tumors in the women from the EPIC and the WHI studies^a^

	Age groups, years							
	40.0–44.9	45.0–49.9	50.0–54.9	55.0–59.9	60.0–64.9	65.0–69.9	70.0–74.9	≥ 75.0
EPIC study								
ER+	30	88	152	189	227	239	233	186
ER−	19	35	44	43	42	41	40	31
WHI study								
ER+	–	–	126	173	185	237	243	270
ER−	–	–	48	50	43	50	39	50

Hazards are presented as number of tumors per 10^5^ person-years

*EPIC* European Prospective Investigation into Cancer and Nutrition, *WHI* Women’s Health Initiative, *ER* estrogen receptor

^a^Risk factor profiles for baseline hazard: country = France (only for EPIC), menopausal status = 1 (postmenopausal), age at menopause ≤ 45 years, duration of breastfeeding = 0, age at menarche ≤ 11 years, duration of hormone replacement therapy = 0, full-term pregnancy = 0 (nulliparous), body mass index < 25 kg/m^2^, height_d10 (body height in cm divided by 10) = 16, alcohol intake (drinks/day) = 0

## Abbreviations

BC: Breast cancer
BMI: Body mass index
CI: Confidence interval
EPIC: European Prospective Investigation into Cancer and Nutrition
E/O: Ratio of observed to expected
ER: Estrogen receptor
FHBC: Family history of breast cancer
FTP: Full-term pregnancy
HR: Hazard ratio
HRT: Hormone replacement therapy
IRS: Immunoreactive score
Model_ER-_: Risk prediction model of estrogen-negative tumors
Model_ER+_: Risk prediction model of estrogen-positive tumors
Model_Gail_: Gail model for breast cancer risk
PR: Progesterone receptor
WHI: Women’s Health Initiative
